# Vancomycin population pharmacokinetics in patients with burns

**DOI:** 10.3389/fmed.2026.1829805

**Published:** 2026-06-22

**Authors:** Yan Shi, Yijin Yao, Xiaoshuang He, Xiaolan Bian, Xunben Yu, Jie Fang, Huan Wang, Yi Dou

**Affiliations:** 1 Department of Burn, Shanghai Burn Institute, Ruijin Hospital, Shanghai Jiao Tong University School of Medicine, Shanghai, China; 2 Department of Respiratory and Critical Care Medicine, School of Medicine, Ruijin Hospital, Shanghai Jiao Tong University, Shanghai, China; 3 Department of Pharmacy, School of Medicine, Ruijin Hospital, Shanghai Jiao Tong University, Shanghai, China; 4 Department of Pharmacy, Ruijin Hospital Luwan Branch, Shanghai Jiao Tong University School of Medicine, Shanghai, China; 5 Department of Pharmacy, The First Affiliated Hospital of Wenzhou Medical University, Wenzhou, China; 6 Department of Pharmacy, The Fifth Affiliated Hospital, Xinjiang Medical University, Ürümqi, China

**Keywords:** burn, creatinine clearance, individualized medication, PPK model, vancomycin

## Abstract

**Background:**

Vancomycin is used to treat severe infections caused by methicillin-resistant gram-positive bacteria such as methicillin-resistant *Staphylococcus aureus* (MRSA). Although several pharmacokinetic studies on vancomycin in burn patients have been reported, a robust population pharmacokinetic model based on routine therapeutic drug monitoring data from a large cohort is still lacking. This study aimed to develop a PopPK model for vancomycin in adult burn patients and evaluate its predictive performance.

**Methods:**

Serum vancomycin concentrations from 93 burn patients were analyzed using non-linear mixed-effects modeling. Covariates including age, gender, creatinine clearance (CrCL), BMI, burn cause, and burn area were evaluated using stepwise forward selection and backward elimination. Model stability was assessed by bootstrap. Predictive performance was compared with Clincalc in 9 external burn patients.

**Results:**

A one-compartment model best described vancomycin PK in burn patients. The final model identified CrCL as a significant covariate on clearance: CL (L/h) = 6.73 × (CrCL/111.67)^0.708^, with a volume of distribution of 49.1 L. Bootstrap analysis confirmed good model stability. Our model demonstrated superior predictive accuracy compared with Clincalc.

**Conclusion:**

Vancomycin clearance is increased in burn patients and significantly correlated with CrCL. The developed PopPK model provides a reliable basis for individualized vancomycin dosing in this specific population.

## 1 Introduction

Burn wounds destroy the skin barrier against microbial invasion, and necrotic tissue provides a favorable environment for the growth of microorganisms (1–3). Therefore, it is difficult to avoid wound infections in patients with burns. Wound infections are difficult to avoid in burn patients. Therefore, prompt isolation and identification of the causative organism are essential to guide appropriate antibiotic therapy, as delayed or ineffective treatment may lead to serious systemic infection. A series of pathological and physiological processes occur after burns, causing changes in capillary permeability and glomerular filtration rate; these may alter the pharmacokinetics of antibiotics *in vivo* (4, 5). Moreover, the administration of high volumes of intravenous fluids during the volume resuscitation phase after severe burns can also affect antibiotic pharmacokinetics, making effective treatment with antibiotics more difficult in the early post-burn period, during which routine systemic antimicrobial prophylaxis is not recommended unless infection is documented. Therefore, given the pathological and physiological changes in patients with severe burns, the use and dosage of antibiotics may need to be adjusted; this requires the application of pharmacokinetic methods to guide clinical strategies. The incidence of multidrug-resistant bacteria, particularly methicillin-resistant *Staphylococcus aureus* (MRSA), is increasing in burn wards, and vancomycin is the first-choice antibiotic for treating MRSA infection (2, 3). Vancomycin works by inhibiting the synthesis of the bacterial cell wall, altering the permeability of the bacterial cell membrane and hindering the synthesis of bacterial RNA (6). Based on pharmacokinetic/pharmacodynamic (PK/PD) principles and clinical studies, the suggested target trough concentration to minimize toxicities and maximize efficacy is 10–20 mg/L (7, 8). While trough concentrations have been historically used, current guidelines increasingly recommend AUC-based exposure metrics to optimize efficacy and minimize toxicity. This recommended dosing regimen and concentration range has been determined via pre-clinical studies in healthy and young volunteers. However, vancomycin pharmacokinetics varies substantially across different populations. Although several clinical decision support systems and published pharmacokinetic studies have addressed vancomycin use in burn patients, these existing models were primarily developed from non-burn populations or small cohorts and do not adequately capture burn-specific pathophysiological alterations, such as altered capillary permeability, augmented renal clearance, large-volume fluid resuscitation, and potential extra-renal drug losses through open wounds. When applied to burn patients, these models often yield suboptimal predictions, as evidenced by the discrepancies between predicted and measured concentrations in our clinical practice. This highlights the need for a robust, burn-specific population PK model derived from a larger cohort to improve dosing accuracy in this population (9–11). Although several pharmacokinetic studies on vancomycin in burn patients have been reported, there are currently no specific clinical guidelines for vancomycin dosing regimens in this population. Addressing this gap, this study collected data from patients with burns treated with vancomycin, and developed pharmacokinetic models for comparison with existing ones, aiming to improve the effective use of vancomycin in this patient group.

## 2 Materials and methods

### 2.1 Patients and study design

This study was a pharmacokinetic, prospective, open label trial. It included patients with burns who had been admitted to the Department of Burns from 2017 to 2019 in the Ruijin Hospital of Shanghai, China. The Ethical Committee for Research approved this prospective study (approval number: M012017), and required signed written informed consent from each patient. All patient data and samples were obtained in strict accordance with the approved study protocol and ethical requirements. The enrollment criteria were as follows: (1) Admitted to the hospital with burns; (2) ≥ 18 years old; (3) Vancomycin was used, and the time of administration and sampling were recorded accurately. The exclusion criteria were as follows: (1) having serious adverse drug reactions; and/or (2) requiring renal replacement therapy while receiving vancomycin. The body weight, height, gender, age, creatinine, burned area, vancomycin dosage, administration time, serum concentration and cause of injury (flame/scald/chemical/contact/electrical) of the patients were collected and recorded.

### 2.2 Blood sampling and vancomycin assays

Patients received vancomycin by intermittent intravenous infusion over 3 h. A fixed dosing regimen of 1 g every 12 h was employed, and no loading doses were administered. Steady state was assumed to be achieved after five doses. Blood samples were collected half an hour before (trough concentration) and one hour after the end (peak concentration) of administration. All administration times and sampling times were accurately recorded in the medical chart for PK analysis. Other information such as creatinine clearance was recorded based on the actual test date.

Vancomycin serum concentrations were measured using an “Abbott i2000” assay system based on immunoassay, following the manufacturer’s procedure. The quantification range is 0.24–100 mg/L.

### 2.3 Population pharmacokinetic analysis

PPK modeling was performed using non-linear mixed-effects analysis with NONMEM (version 7.4, Icon Development Solutions, Ellicott City, MD, USA), Pirana (version 2.9.7), R (version 3.6.0) and Xpose (version 4.3.2) software packages were applied to generate diagnostic plots.

We used a one-compartment, open kinetic model with first-order conditional estimation (specified to NONMEM by the ADVAN1 and TRANS2 routines) for estimates for vancomycin clearance (CL) and volume of distribution (V).

A power model was used to estimate the variation in PK parameters between individuals. The residual variation was assessed by the additive model, proportional model, and mixed model. Covariate analysis was performed by a stepwise forward selection and backward elimination procedure.

The potential parameters including age, gender, serum creatinine concentration, and BMI were introduced into the PK model by linear, allometric or exponential functions.

In the stepwise forward inclusion procedure, a covariate was included in the model if the objective function value (OFV) was significantly (*p* < 0.05) decreased relative to the base model (OFV reduction > 3.84). All statistically significant covariates were added to the basic model to create a full model. In the backward elimination procedure, the covariates were refiltered by the criterion about *p* < 0.001 (OFV increase > 10.83).

### 2.4 Model verification

Several graphics and statistics were performed to evaluate the final model, including goodness-of-fit plots and non-parametric bootstrap. Goodness-of-fit was evaluated using diagnostic scatter plots, including observed concentrations vs. population predicted concentrations, observed concentrations vs. individual predicted concentrations, conditional weighted residuals vs. population predicted concentrations and conditional weighted residuals vs. time. The stability of the final model was evaluated by the non-parametric bootstrap procedure with random sampling and replacement. The random sampling was repeated 1,000 times. The median and 95% confidence interval values of estimated parameters from the bootstrap procedure were compared with the final parameters estimates from the NONMEM program.

## 3 Results

### 3.1 Baseline characteristics of patients

A total of 93 patients (60 male and 33 female) were evaluated in this PPK model study, and included in final pharmacokinetic analysis. All patients had at least one vancomycin steady-state trough or peak serum concentration. For all subjects, concentrations were obtained after at least three doses of vancomycin. The 93 patients had a total of 183 vancomycin serum concentrations measured. Parameter estimates from the base model are provided in Supplementary Table 1. Other patient features are listed in Table 1.

**TABLE 1:** Demographic and clinical characteristics of 93 patients.

Parameters		Values
Male		63 (67.74%)
Age (years)		44.04 ± 13.94
BMI		23.54 ± 3.28
Creatinine clearance (mL/min)		116.82 ± 40.38
Usage and dosage		1 g q12 h
Burn cause	Flame	59 (63.44%)
	Chemical agents	4 (4.30%)
	Electrical shock	8 (8.60%)
	Hydrothermal solution	15 (16.13%)
	Heating power	7 (7.53%)
Burn surface (%TBSA)	< 30%	51 (54.84%)
	30%–50%	15 (16.13%)
	> 50%	27 (29.03%)
Third degree burn areas	< 2%	34 (36.56%)
	2%–10%	25 (26.88%)
	> 10%	34 (36.56%)

### 3.2 Population pharmacokinetic modeling analysis

These concentration values ranged from 0.1 to 46.2 mg/L and were included in the pharmacokinetic analysis. The PK characteristics of vancomycin in the patients with burns could be characterized by a one-compartment model, with linear elimination showing the best fit of the observed concentration-versus-time data based on the reduction in OFV. Parameter estimates from the base model are provided in Supplementary Table 1.

The final model was selected by the stepwise forward selection and reverse elimination process of covariates; the results are provided in Supplementary Tables 2, 3.

Minimization and the covariance step were successful in the final model. Table 2 lists the estimate, RSE and residual variability of the parameters for the final model. These estimates demonstrated an acceptable precision (RSE% < 30%). In the final model, the typical value of typical value of clearance (TVCL) and typical value of apparent volume of distribution (TVV) was 6.73 L/h and 49.1 L, respectively. The final model is listed below:


V⁢(L)=49.1⁢L (1)

$$C\,L\,\left(\frac{L}{h}\right)=6.73\times {\left(\frac{C\,r\,C\,L}{111.67}\right)}^{0.708}$$ (2)

**TABLE 2:** Population pharmacokinetic parameter estimates from the final model.

Parameter	Estimate	RSE	Shrinkage
Fixed effects			
TVCL[L/h]	6.73	4%	
TVV[L]	49.1	5%	
CrCL on CL	0.708	14%	
Between-subject variability (BSV)			
BSV_CL [%CV]	37.90%	12%	7%
BSV_V [%CV]	36.20%	17%	24%
Residual variability (RV)			
Proportional error [%CV]	14.1%	19%	49%

Symbols in Equations 1, 2 are as follows: CL is the individual clearance, V is the individual volume of distribution, creatinine clearance (CrCL) is the estimated creatinine clearance.

**FIGURE 1:**
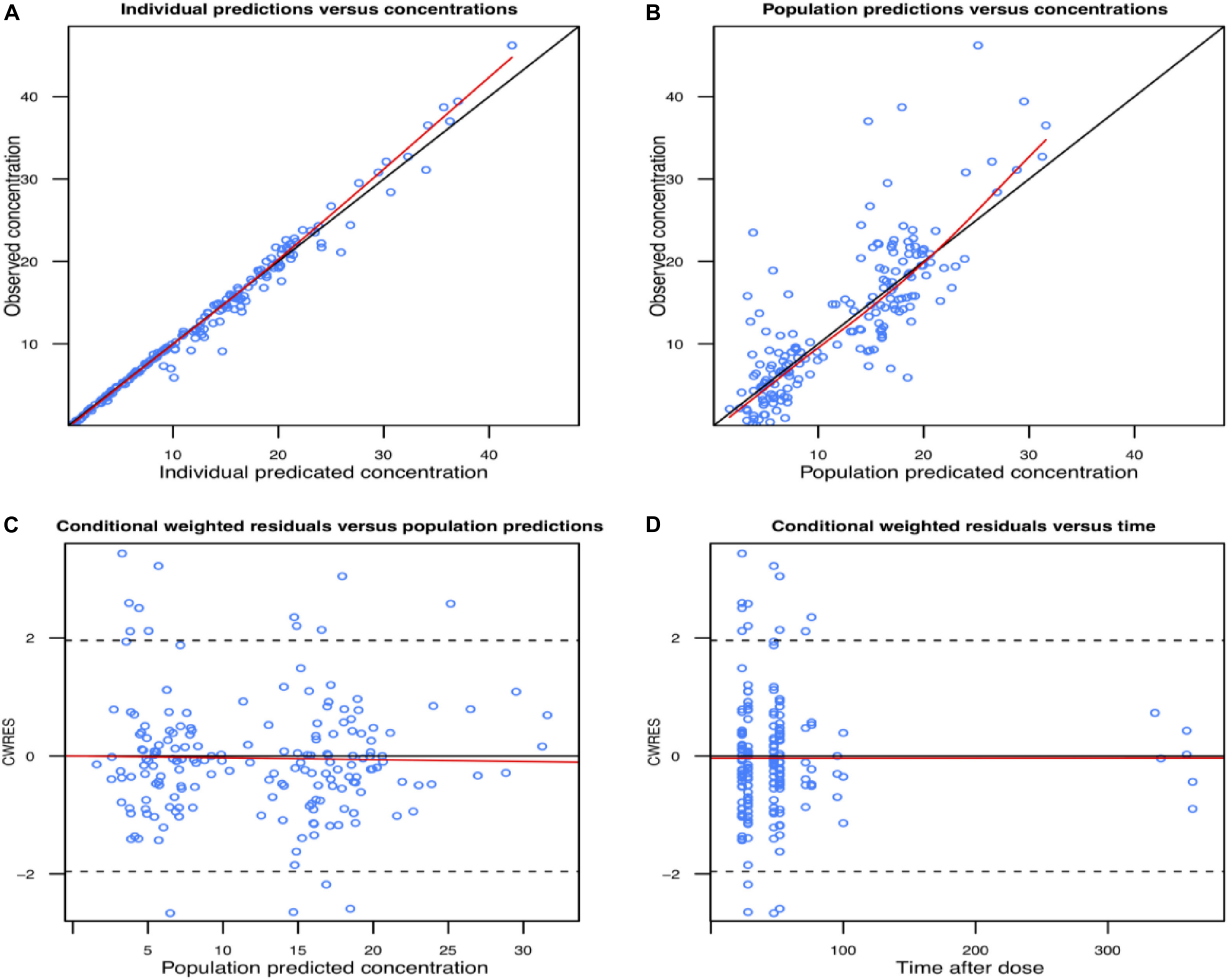
Goodness-of-fit in the final model. **(A)** Observed concentration versus individual predicted concentration; **(B)** observed concentration versus population predicted concentration; **(C)** conditional weighted residuals versus population predicted concentration; and **(D)** conditional weighted residuals versus time. The red lines in the upper panel represent lines and linear fit lines.

The diagnostic plots to confirm the goodness-of-fit from the final covariate model were considered acceptable and displayed in Figure 1. The conditional weighted residuals versus the population predicted concentration of the final model showed a random distribution near zero; most values were within an acceptable range of ± 2. The bootstrap verification results are shown in Table 3. The results showed that not only did the parameter estimates of the final model lie within the 95% CI resulted from the non-parametric bootstrap procedure, but the biases between the final model estimates and bootstrapped median parameter estimates were < ± 5% for all parameters, indicating the good stability of the final model. Overall, these results show that the established vancomycin population PK model provides sufficient description of data and good prediction of individual PK parameters.

**TABLE 3:** Vancomycin PopPK parameter estimates of the final model and bootstrap results.

Parameter	Final model		Bootstrap results			Bias (%)
	Estimate	RSE (%)	2.5th percentile	Median estimate	97.5th percentile	
θ_*CL*_ [L/h]	6.73	4	6.23	6.73	7.27	0.00
θ_*V*_ [L]	49.1	5	44.78	49.09	53.74	−0.02
θ_1_	0.708	14	0.49	0.71	0.91	0.28
Inter-individual variability						
ω_*CL*_ [%]	37.90	12	28.00	37.48	46.72	−1.11
ω_*V*_ [%]	36.20	17	17.09	35.83	47.14	−1.02
Residual variability						
σ_*pro*_ (%)	14.10	19	7.22	14.16	20.63	0.43

PopPK, population pharmacokinetic; RSE (%), relative standard error; θ_*CL*_, typical value of apparent clearance; θ_*V*_, typical value of apparent volume of distribution; θ_1_, allometric value for CrCL as covariate for CL; ω_*CL*_, square root of inter-individual variance for CL; ω_*V*_, square root of inter-individual variance for V; σ_*pro*_, residual variability for proportional error. Bias (%) = (Median Estimate_*Bootstrap*_−Estimate_*Final model*_)/Estimate_*Final model*_ × 100%.

### 3.3 Comparison with Clincalc results

In recent years, an individualized drug administration decision system based on PPK and Bayesian algorithms has been widely used in clinical practice. Smart Dose, Pharm Van and Clincalc can all provide individualized dosing regimens for vancomycin. However, they are not suitable for the formulation and prediction of dosing regimens in patients with burns. Based on our analysis, clinical decision support systems such as Smart Dose, Pharm Van, and Clincalc are not suitable for burn patients. Their embedded PK models were developed primarily from general critically ill patients or healthy volunteers and do not account for burn-specific physiological alterations, including increased capillary permeability, augmented renal clearance, potential extra-renal drug losses through burn wounds, and large-volume fluid resuscitation. These burn-specific factors result in significantly altered vancomycin clearance compared to standard populations, leading to suboptimal predictions when generic models are applied.

We selected-external data from nine patients with burns who were treated with vancomycin. Their peak and trough concentrations were collected and compared with the prediction results obtained using our established model and Clincalc. From the results (Table 4), it can be seen that the prediction results of our established model are closer to the real measured values.

**TABLE 4:** The comparison results of vancomycin concentration.

Observed concentration (mg/L)		Predicted concentration (mg/L)		Prediction error (%)^a^	
No.	Tough concentration	Final model	Clincalc	Final model	Clincalc
1	5.4	8.46	20.9	56.67	287.04
2	5.9	3.75	14.9	−36.44	152.54
3	6.7	4.97	13.4	−25.82	100.00
4	9.2	8.07	18.9	−12.28	105.43
5	10.1	13.42	49.7	32.87	392.08
6	8.4	13.57	42.5	61.55	405.95
7	6.9	6.56	16.3	−4.93	136.23
8	20.4	16.57	42.5	−18.77	108.33
9	29.5	21.33	221.8	−27.69	651.86
**No.**	**Peak concentration**	**Final model**	**Clincalc**	**Final model**	**Clincalc**
1	19.3	20.46	27.7	6.01	43.52
2	11.5	14.91	21	29.65	82.61
3	15.6	16.26	19.9	4.23	27.56
4	18.5	19.82	25.9	7.14	40.00
5	26.9	37.14	62.1	38.07	130.86
6	40.8	37.29	56.5	−8.60	38.48
7	22.9	18.4	24.1	−19.65	5.24
8	28.2	30.22	55.5	7.16	96.81
9	41.1	34.88	239.7	−15.13	483.21

^a^Prediction error (%) = Predicted concentration-Observed concentration /Observed concentration × 100.

## 4 Discussion

Vancomycin pharmacokinetics demonstrates high interindividual variability in burn patients compared to other populations, which indicates the importance of adjusting vancomycin dose in the former in order to optimize vancomycin concentration.

To date, the high inter-patient variability of vancomycin pharmacokinetics in patients with thermal injury has been widely documented but insufficiently explained. In this study, we attempted to identify more variables to reflect the physiological and pathological changes that influence vancomycin pharmacokinetics in patients with burns. The use of estimated CrCL as a reliable drug delivery tool for vancomycin in critically ill patients has been validated and rejected (12–14). As Elder et al. (15) mentioned, the accuracy of the CrCL equation in predicting the true filtration ability of the kidney in patients with burns has been questioned. This criticism might be valid. Because of the initial study, women were largely under-represented limiting the applicability of the derived equation and correction factor (16). However, vancomycin showed renal tubular secretion in addition to glomerular filtration, which might not be proportional to CrCL during acute disease (14). Large open wounds seen in burn injuries could also potentially account for extra-renal losses. Burns cause widespread skin and tissue damage and release of systemic inflammatory mediators, which promote endothelial leakage, extravascular fluid shifts, and cardiovascular derangement. The development of relative intra-vascular hypovolaemia leads to altered peripheral tissue perfusion, and large-volume intravenous fluid resuscitation is administered during this phase, ultimately resulting in haemodynamic perturbation.

Several studies about the pharmacokinetics of vancomycin in patients with burns have been reported, as summarized in Table 5. Dolton et al. (17) reported that CL in these patients (5.9 L/h) was remarkably higher than in control patients (3.4 L/h), which might be correlated with CrCL in the former. Similarly, multiple studies of vancomycin in such patients have documented a significant association between CrCL and CL (13, 18, 19). The results showed that the pharmacokinetic parameters of vancomycin, such as its clearance, were altered in these patients. However, only a small number of cases was included. To explore the variation in pharmacokinetic parameters in patients with burns, a total of 93 patients, comprising 60 males and 33 females, were included in this study.

**TABLE 5:** Summary of pharmacokinetic studies on vancomycin for burn patients.

References	*N*	Age (years)	%TBSA	CrCL (mL/min)	Vancomycin CL (L/h)
Present study	93	45.03 ± 14.28	35.10 ± 28.75	120.32 ± 43.72	6.73 ± 3.08
Yang et al. (28)	10	30.54 ± 8.04	64.65 ± 13.59	95.45 ± 23.14	NR
Dolton et al. (17)	37	34 (15–88)^a^	≥ 10^b^	124 ± 56	5.90 ± 3.10
Brater et al. (12)	11	41.00 ± 4.00	50.00 ± 3.00	105 ± 19	5.60 ± 3.40
Garrelts and Peterie (24)	9	28.00 ± 13.00	24.00 ± 14.10	131 ± 25	5.80 ± 1.70
Rybak et al. (13)	10	36.00 ± 15.00	> 10^b^	111 ± 28	8.60 ± 2.10^c^
Zokufa et al. (29)	10	54.00 ± 19.00	29.00 ± 16.00	100 ± 11	NR

NR, information not recorded.

^a^Median (range) for burns patients in the study;

^b^no specific data provided in the study;

^c^calculated estimate for a 70 kg patient using mean vancomycin CL values.

The demographic and pharmacokinetic characteristics of the patients in this study are displayed in Table 1. The median time between injury and vancomycin treatment (days since injury, DSI) was 4 days. While most patients were treated more than 48 h after burn, six patients received vancomycin within 48 h post-burn, a period during which the hypermetabolic phase predominates. We acknowledge that the hypermetabolic phase can produce physiological changes—such as fever, tachycardia, and leukocytosis—that may mimic infection (20–23). Therefore, to ensure clinical rigor, all patients in this study had confirmed infection based on positive bacterial cultures or definitive clinical infection criteria prior to vancomycin initiation. Moreover, the hypermetabolic phase is associated with increased cardiac output and augmented renal clearance, which can significantly elevate vancomycin elimination. These further underscores the necessity of close therapeutic drug monitoring during this early post-burn period to ensure adequate drug exposure. The burn cause was almost universally found to be thermal damage, mainly (63.44%) due to flame exposure. Bacterial culture before treatment revealed mainly gram-positive bacteria and culture after treatment mainly comprised gram-negative bacteria.

In addition, some studies have found a strong correlation between vancomycin elimination and CrCL, but others have identified large increases in vancomycin elimination independent of CrCL (24, 25). CrCL might be an important covariate affecting CL. In addition to CrCL, the third-degree burn areas might be another factor affecting CL of vancomycin. The period more than 48 h post-burn corresponds to the hypermetabolic phase of burn injury, which is driven by the systemic effects of inflammation and oxidative stress. At this stage, after sufficient fluid was given, the hemodynamic state of the patient was highly dynamic, with increased cardiac contractility, high cardiac output and systemic vascular resistance. Increased local blood flow, especially in the liver and kidney, leads to increased glomerular filtration rate (GFR) and CrCL. Non-renal clearance rate (CLNR) also increases owing to leakage of exudate from partial and third-degree burn areas, but its importance in drug clearance remains controversial (26). Jeschke et al. (27) reported that animal experiments *in vivo* revealed that burn not only caused liver dysfunction and apoptosis, but also severe calcium homeostasis disorder in liver. Similarly, Bloedow et al. (20) reported that albumin concentrations in patients with burns were lower than the protein concentrations in control subjects during the acute phase of recovery from the burn injury. Bradley et al. (21) reported that 70.3% of hospitals did not make the switch to implement vancomycin AUC dosing in the United States. Similarly, a cross-sectional survey of a national health consortium showed that fewer than one-fourth of hospitals performed AUC-based vancomycin monitoring, with the most common challenge and barrier to implementation of this monitoring strategy being pharmacist and/or provider unfamiliarity (22). In addition, Aljutayli et al. (23) reported that Bayesian-guided AUC may be superior to equation-based AUC prediction according to the quality of model diagnosis and the assumptions satisfied.

In this study, gender, burn causes, age, DSI, CrCL, BMI, and area were shown to be significant covariate of vancomycin exposure. During the forward inclusion step of the covariate modeling process, CrCL and burn cause showed a correlation with the interindividual variation of CL and BMI showed a correlation with the interindividual variation of V. Eventually, only CrCL on CL was retained in the final model. Others were excluded as their OFV decrease in the backward elimination procedure was less than 10.83.

There are some limitations in our study; these are as follows. First, although we performed a preliminary external evaluation using nine independent burn patients to compare our model’s predictive accuracy against Clincalc, the validation sample size was limited. We were therefore unable to conduct a formal external validation with adequate statistical power. We will continue to collect additional independent samples to further validate the predictive capacity of the final model in future studies. Moreover, the variables collected in this study were limited and could not fully reflect the factors influencing individual pharmacokinetic variation of vancomycin. We will continue to enroll additional patients and collect more comprehensive clinical variables to further optimize and validate the model in future studies.

## 5 Conclusion

In conclusion, given the increased clearance and substantial interindividual variability of vancomycin in patients with burns, close therapeutic drug monitoring with appropriate individualized dosing is recommended from the initiation of therapy to ensure adequate drug exposure, rather than waiting until subtherapeutic serum concentrations are identified. The main factors affecting AUC attainment are creatinine clearance and burn area. The population pharmacokinetic model developed herein provides a reliable basis for optimizing vancomycin dosing in this specific patient population.

## Data Availability

The raw data supporting the conclusions of this article will be made available by the authors, without undue reservation.
