# Supplementary material for: Vancomycin population pharmacokinetics in patients with burns
Source: Front Med (Lausanne). 2026 Jun 22;13:1829805. doi: 10.3389/fmed.2026.1829805 (PMC13333703; doi:10.3389/fmed.2026.1829805)
Supplement: Supplementary file 1 [file Table_1.docx]

**Table S1.** Population pharmacokinetic parameter estimates from the base model

| **Parameter** | **Estimate** | **RSE** | **shrinkage** |
| --- | --- | --- | --- |
| **Fixed Effects** |  |  |  |
| TVCL[L/h] | 6.76 | 5% |  |
| TVV[L] | 49.1 | 4% |  |
| **Between-subject Variability (BSV)** |  |  |  |
| BSV_CL [%CV] | 50.60% | 9% | 5% |
| BSV_V [%CV] | 37.90% | 16% | 21% |
| **Residual Variability (RV)** |  |  |  |
| Proportional Error [%CV] | 12.3% | 20% | 53% |

a. BSV calculated as $\sqrt{e^{\omega^{2}}-1}$
