# Supplementary material for: Vancomycin population pharmacokinetics in patients with burns
Source: Front Med (Lausanne). 2026 Jun 22;13:1829805. doi: 10.3389/fmed.2026.1829805 (PMC13333703; doi:10.3389/fmed.2026.1829805)
Supplement: Supplementary file 2 [file Table_2.docx]

**Table S2.** Forward selection of covariates

| **PAR** | **COV** | **Functional Form** | **DF** | **Min,ROUND, &COVAR Successful** | **OFV** | **AIC** | **BSV_CL** | **BSV_V** |
| --- | --- | --- | --- | --- | --- | --- | --- | --- |
| **Round 1 (Reference Model: Base)** | | | | | | | | |
| **REF** | **--** | **--** | **--** | **--** | **739.475** | **749.475** | **50.6%** | **37.9%** |
| CL | GEND | Add Shift | 1 | YES | 738.56 | 750.56 | 50.0% | 37.9% |
| CL | REASON | Add Shift | 1 | YES | 735.931 | 747.931 | 49.5% | 36.5% |
| CL | AGE | Linear | 1 | YES | 720.9 | 732.9 | 44.8% | 37.9% |
| CL | AGE | Allometric | 1 | YES | 722.745 | 734.745 | 45.3% | 37.6% |
| CL | AGE | Exponent | 1 | YES | 721.362 | 733.362 | 44.9% | 37.7% |
| CL | DSI | Linear | 1 | YES | 736.158 | 748.158 | 49.2% | 38.0% |
| CL | DSI | Allometric | 1 | NO | / | / | / | / |
| CL | CRCL | Linear | 1 | YES | 699.351 | 711.351 | 38.5% | 36.3% |
| **CL** | **CRCL** | **Allometric** | **1** | **YES** | **697.188** | **709.188** | **36.9%** | **36.2%** |
| CL | CRCL | Exponent | 1 | YES | 706.675 | 718.675 | 40.1% | 36.3% |
| CL | BMI | Linear | 1 | YES | 734.78 | 746.78 | 48.8% | 38.2% |
| CL | BMI | Allometric | 1 | YES | 734.87 | 746.87 | 48.8% | 38.2% |
| CL | BMI | Exponent | 1 | YES | 734.507 | 746.507 | 48.7% | 38.3% |
| CL | AREA | Linear | 1 | YES | 738.836 | 750.836 | 50.5% | 37.9% |
| CL | AREA | Allometric | 1 | YES | 737.863 | 749.863 | 50.0% | 37.3% |
| CL | AREA | Exponent | 1 | YES | 738.895 | 750.895 | 50.5% | 37.9% |
| V | GEND | Add Shift | 1 | YES | 736.694 | 748.694 | 50.7% | 37.4% |
| V | REASON | Add Shift | 1 | YES | 734.436 | 746.436 | 50.9% | 35.9% |
| V | AGE | Linear | 1 | YES | 739.363 | 751.363 | 50.5% | 37.9% |
| V | AGE | Allometric | 1 | NO | / | / | / | / |
| V | AGE | Exponent | 1 | YES | 739.368 | 751.368 | 50.5% | 37.9% |
| V | DSI | Linear | 1 | YES | 735.867 | 747.867 | 49.2% | 38.0% |
| V | DSI | Allometric | 1 | NO | / | / | / | / |
| V | DSI | Exponent | 1 | NO | / | / | / | / |
| V | CRCL | Linear | 1 | YES | 739.146 | 751.146 | 50.7% | 37.9% |
| V | CRCL | Allometric | 1 | YES | 738.525 | 750.525 | 50.7% | 37.7% |
| V | CRCL | Exponent | 1 | YES | 739.181 | 751.181 | 50.7% | 37.9% |
| V | BMI | Linear | 1 | YES | 734.441 | 746.441 | 51.1% | 37.1% |
| V | BMI | Allometric | 1 | YES | 734.488 | 746.488 | 51.1% | 48.5% |
| V | BMI | Exponent | 1 | YES | 734.326 | 746.326 | 51.1% | 37.1% |
| V | AREA | Linear | 1 | YES | 739.404 | 751.404 | 50.6% | 37.9% |
| V | AREA | Allometric | 1 | YES | 735.711 | 747.711 | 50.7% | 36.5% |
| V | AREA | Exponent | 1 | YES | 739.41 | 751.41 | 50.6% | 37.9% |

| **Round 2 (Reference Model: Base+CRCL_CL)** | | | | | | | | |
| --- | --- | --- | --- | --- | --- | --- | --- | --- |
| **REF** | **--** | **--** | **--** | **--** | **697.188** | **709.188** | **36.9%** | **36.2%** |
| CL | GEND | Add Shift | 1 | YES | 697.188 | 711.188 | 37.9% | 36.2% |
| CL | REASON | Add Shift | 1 | YES | 692.608 | 706.608 | 37.0% | 35.5% |
| CL | AGE | Linear | 1 | YES | 696.543 | 710.543 | 37.9% | 36.5% |
| CL | AGE | Allometric | 1 | YES | 696.611 | 710.611 | 37.9% | 36.3% |
| CL | AGE | Exponent | 1 | YES | 696.825 | 710.825 | 37.9% | 36.3% |
| CL | DSI | Linear | 1 | YES | 692.794 | 706.794 | 36.6% | 36.2% |
| CL | DSI | Allometric | 1 | NO | / | / | / | / |
| CL | BMI | Linear | 1 | YES | 696.711 | 710.711 | 37.9% | 36.2% |
| CL | BMI | Allometric | 1 | YES | 697.154 | 711.154 | 37.9% | 36.2% |
| CL | BMI | Exponent | 1 | YES | 697.184 | 711.184 | 37.9% | 36.2% |
| CL | AREA | Linear | 1 | YES | 695.099 | 709.099 | 37.4% | 36.2% |
| CL | AREA | Allometric | 1 | YES | 697.097 | 711.097 | 37.9% | 36.2% |
| CL | AREA | Exponent | 1 | YES | 694.677 | 708.677 | 37.4% | 36.3% |
| V | GEND | Add Shift | 1 | YES | 694.764 | 708.764 | 37.9% | 35.4% |
| V | REASON | Add Shift | 1 | YES | 692.289 | 706.289 | 37.9% | 33.8% |
| V | AGE | Linear | 1 | YES | 696.677 | 710.677 | 37.7% | 35.9% |
| V | AGE | Allometric | 1 | YES | 696.756 | 710.756 | 37.7% | 36.0% |
| V | AGE | Exponent | 1 | YES | 696.678 | 710.678 | 37.7% | 35.9% |
| V | DSI | Linear | 1 | YES | 696.964 | 710.964 | 38.0% | 36.3% |
| V | DSI | Allometric | 1 | NO | / | / | / | / |
| V | DSI | Exponent | 1 | NO | / | / | / | / |
| V | CRCL | Linear | 1 | YES | 697.167 | 711.167 | 37.9% | 36.2% |
| V | CRCL | Allometric | 1 | YES | 697.054 | 711.054 | 37.9% | 36.2% |
| V | CRCL | Exponent | 1 | YES | 697.169 | 711.169 | 37.9% | 36.2% |
| V | BMI | Linear | 1 | YES | 692.087 | 706.087 | 38.0% | 35.1% |
| V | BMI | Allometric | 1 | YES | 692.139 | 706.139 | 38.0% | 35.1% |
| **V** | **BMI** | **Exponent** | **1** | **YES** | **691.971** | **705.971** | **38.0%** | **35.1%** |
| V | AREA | Linear | 1 | YES | 697.077 | 711.077 | 37.9% | 36.2% |
| V | AREA | Allometric | 1 | YES | 693.681 | 707.681 | 37.7% | 34.6% |
| V | AREA | Exponent | 1 | YES | 697.086 | 711.086 | 37.9% | 36.2% |

| **Round 3 (Reference Model: Base+CRCL_CL+BMI_V)** | | | | | | | | |
| --- | --- | --- | --- | --- | --- | --- | --- | --- |
| **REF** | **--** | **--** | **--** | **--** | **691.971** | **705.971** | **38.0%** | **35.1%** |
| CL | GEND | Add Shift | 1 | YES | 691.966 | 707.966 | 38.0% | 35.1% |
| **CL** | **REASON** | **Add Shift** | **1** | **YES** | **687.451** | **703.451** | **37.1%** | **34.6%** |
| CL | AGE | Linear | 1 | YES | 691.56 | 707.56 | 38.0% | 35.2% |
| CL | AGE | Allometric | 1 | YES | 691.465 | 707.465 | 37.9% | 35.1% |
| CL | AGE | Exponent | 1 | YES | 691.683 | 707.683 | 37.9% | 35.1% |
| CL | DSI | Linear | 1 | YES | 688.288 | 704.288 | 36.8% | 35.1% |
| CL | DSI | Allometric | 1 | NO | / | / | / | / |
| CL | DSI | Exponent | 1 | NO | / | / | / | / |
| CL | BMI | Linear | 1 | YES | 691.781 | 707.781 | 38.0% | 35.1% |
| CL | BMI | Allometric | 1 | YES | 691.963 | 707.963 | 38.0% | 35.1% |
| CL | BMI | Exponent | 1 | YES | 691.917 | 707.917 | 38.0% | 35.1% |
| CL | AREA | Linear | 1 | YES | 689.824 | 705.824 | 37.6% | 35.1% |
| CL | AREA | Allometric | 1 | YES | 691.898 | 707.898 | 38.0% | 34.9% |
| CL | AREA | Exponent | 1 | YES | 689.409 | 705.409 | 37.4% | 35.1% |
| V | GEND | Add Shift | 1 | YES | 690.246 | 706.246 | 37.9% | 34.4% |
| V | REASON | Add Shift | 1 | YES | 690.549 | 706.549 | 38.0% | 34.1% |
| V | AGE | Linear | 1 | YES | 690.68 | 706.68 | 37.7% | 34.4% |
| V | AGE | Allometric | 1 | YES | 690.952 | 706.952 | 37.7% | 34.6% |
| V | AGE | Exponent | 1 | YES | 690.8 | 706.8 | 37.7% | 34.4% |
| V | DSI | Linear | 1 | YES | 691.958 | 707.958 | 38.0% | 35.1% |
| V | DSI | Allometric | 1 | NO | / | / | / | / |
| V | DSI | Exponent | 1 | NO | / | / | / | / |
| V | CRCL | Linear | 1 | YES | 691.066 | 707.066 | 37.9% | 34.9% |
| V | CRCL | Allometric | 1 | YES | 691.593 | 707.593 | 37.9% | 34.9% |
| V | CRCL | Exponent | 1 | YES | 691.083 | 707.083 | 37.9% | 34.9% |
| V | AREA | Linear | 1 | YES | 691.584 | 707.584 | 38.0% | 35.1% |
| V | AREA | Allometric | 1 | YES | 689.388 | 705.388 | 37.9% | 33.6% |
| V | AREA | Exponent | 1 | YES | 691.656 | 707.656 | 38.0% | 35.1% |

| **Round 4 (Reference Model: Base+CRCL_CL+BMI_V+REASON_CL)** | | | | | | | | |
| --- | --- | --- | --- | --- | --- | --- | --- | --- |
| **REF** | **--** | **--** | **--** | **--** | **687.451** | **703.451** | **37.1%** | **34.6%** |
| CL | GEND | Add Shift | 1 | YES | 687.192 | 705.192 | 37.1% | 34.6% |
| CL | AGE | Linear | 1 | YES | 686.957 | 704.957 | 37.1% | 34.9% |
| CL | AGE | Allometric | 1 | YES | 686.095 | 704.095 | 36.8% | 34.7% |
| CL | AGE | Exponent | 1 | YES | 686.383 | 704.383 | 37.0% | 34.7% |
| CL | DSI | Linear | 1 | YES | 683.723 | 701.723 | 35.9% | 35.1% |
| CL | DSI | Allometric | 1 | NO | / | / | / | / |
| CL | DSI | Exponent | 1 | NO | / | / | / | / |
| CL | BMI | Linear | 1 | YES | 687.182 | 705.182 | 37.1% | 34.7% |
| CL | BMI | Allometric | 1 | YES | 687.434 | 705.434 | 37.1% | 34,6% |
| CL | BMI | Exponent | 1 | YES | 687.449 | 705.449 | 37.1% | 34.6% |
| CL | AREA | Linear | 1 | YES | 685.752 | 703.752 | 36.8% | 34.4% |
| CL | AREA | Allometric | 1 | YES | 686.832 | 704.832 | 37.0% | 34.3% |
| CL | AREA | Exponent | 1 | YES | 685.673 | 703.673 | 36.8% | 34.4% |
| V | GEND | Add Shift | 1 | YES | 685.67 | 703.67 | 37.1% | 33.9% |
| V | REASON | Add Shift | 1 | YES | 684.136 | 702.136 | 37.0% | 32.4% |
| V | AGE | Linear | 1 | YES | 686.037 | 704.037 | 36.8% | 33.9% |
| V | AGE | Allometric | 1 | YES | 686.366 | 704.366 | 37.0% | 34.1% |
| V | AGE | Exponent | 1 | YES | 686.179 | 704.179 | 37.0% | 33.9% |
| V | DSI | Linear | 1 | YES | 687.45 | 705.45 | 37.1% | 34.6% |
| V | DSI | Allometric | 1 | NO | / | / | / | / |
| V | DSI | Exponent | 1 | YES | 687.63 | 705.63 | 37.1% | 34.7% |
| V | CRCL | Linear | 1 | YES | 686.356 | 704.356 | 37.0% | 34.4% |
| V | CRCL | Allometric | 1 | YES | 686.941 | 704.941 | 37.1% | 34.4% |
| V | CRCL | Exponent | 1 | YES | 686.391 | 704.391 | 37.0% | 34.4% |
| V | AREA | Linear | 1 | YES | 686.967 | 704.967 | 37.3% | 34.6% |
| V | AREA | Allometric | 1 | YES | 685.27 | 703.27 | 37.1% | 33.4% |
| V | AREA | Exponent | 1 | YES | 687.053 | 705.053 | 37.3% | 34.6% |
