# Supplementary material for: Vancomycin population pharmacokinetics in patients with burns
Source: Front Med (Lausanne). 2026 Jun 22;13:1829805. doi: 10.3389/fmed.2026.1829805 (PMC13333703; doi:10.3389/fmed.2026.1829805)
Supplement: Supplementary file 3 [file Table_3.docx]

**Table S3.** Backward Elimination of covariates

| **PAR** | **COV** | **Functional Form** | **DF** | **Min,ROUND, &COVAR Successful** | **OFV** | **AIC** | **BSC_CL** | **BSV_V** |
| --- | --- | --- | --- | --- | --- | --- | --- | --- |
| **Round 1 (Reference Model=Base+CRCL_CL+BMI_V+REASON_CL)** | | | | | | | | |
| **REF** | **--** | **--** | **--** | **--** | **687.451** | **703.451** | **37.10%** | **34.60%** |
| CL | CRCL | Allometric | 1 | YES | 730.769 | 744.769 | 50.00% | 35.70% |
| V | BMI | Exponent | 1 | YES | 692.608 | 706.608 | 37.00% | 35.50% |
| **CL** | **REASON** | **Add Shift** | **1** | **YES** | **691.971** | **705.971** | **38.00%** | **35.10%** |
| **Round 2 (Reference Model=Base+CRCL_CL+BMI_V)** | | | | | | | | |
| **REF** | **--** | **--** | **--** | **--** | **691.971** | **705.971** | **38.00%** | **35.10%** |
| CL | CRCL | Allometric | 1 | YES | 734.326 | 746.326 | 50.00% | 35.70% |
| **V** | **BMI** | **Exponent** | **1** | **YES** | **697.188** | **709.188** | **37.90%** | **36.20%** |
| **Round 3 (Reference Model=Base+CRCL_CL)** | | | | | | | | |
| **REF** | **--** | **--** | **--** | **--** | **697.188** | **709.188** | **38.00%** | **35.10%** |
| CL | CRCL | Allometric | 1 | YES | 739.475 | 749.475 | 50.60% | 37.90% |
